# Breast Cancer Risk Among Women in Jail

**DOI:** 10.1089/biores.2018.0018

**Published:** 2018-09-20

**Authors:** Michelle L. Pickett, Molly Allison, Katelyn Twist, Jennifer R. Klemp, Megha Ramaswamy

**Affiliations:** ^1^ Section of Emergency Medicine, Department of Pediatrics, Medical College of Wisconsin, Milwaukee, Wisconsin.; ^2^ Department of Preventive Medicine and Public Health, University of Kansas Medical Center, Kansas City, Kansas.; ^3^ Department of Medical Oncology, University of Kansas Medical Center, Kansas City, Kansas.

**Keywords:** breast cancer, incarceration, jail, mammogram

## Abstract

Over 200,000 women are diagnosed with breast cancer each year, and incarcerated women face unique risks associated with poor access to healthcare. Regular mammography can diagnose breast cancer early, giving the patient the best chance of survival. The objective of this study was to determine the proportion of jail incarcerated women who have received a mammogram and were up-to-date based on the most recent United States Preventive Services Task Force recommendations. This was a secondary analysis of data collected among jailed women who participated in a cervical cancer literacy program. Rates of mammography were calculated for the group overall and for those women 50 years or older. Subgroups were compared using chi-squared tests. Two hundred sixty-one women were included in the analysis, of which 42.1% (*N* = 110) had ever had a mammogram. Of women 50 years old or older (*N* = 28), 75.0% had ever received a mammogram, yet only 39.3% were up-to-date (within the past 2 years). Factors associated with up-to-date mammography included being up-to-date on cervical cancer screening (76.9%) compared with women who were not up-to-date on cervical cancer screening (12.5%), *p* < 0.01, and women experiencing intimate partner violence (IPV) in the past year (71.4%) compared with women with no IPV in the past year (14.2%), *p* = 0.02. The low rates of up-to-date mammography highlight the need for more breast cancer prevention programming among women with criminal justice histories.

## Introduction

Disparities in breast cancer screening, diagnosis, and outcomes persist among American women. Each year, there are about 237,000 new cases of breast cancer diagnosed in women in the United States and about 41,000 breast cancer deaths among women.^1^ While white women do have a slightly higher incidence of breast cancer, African Americans have the highest rate of breast cancer deaths.^1^ It is believed that African Americans are less likely to undergo screening mammography due to worse access in care.^2^ Therefore, breast cancer in African Americans is often diagnosed at later stages, thus making it harder to treat and leading to higher mortality.^3–5^ In addition, socioeconomic disparities faced by African American may lead to worse access and gaps in healthcare impacting breast cancer survival.^6–8^

If breast cancer is diagnosed early through regular screening, such as screening mammography, the result is better outcomes and a more favorable prognosis. The United States Preventive Services Task Force (USPSTF) has recommended biennial screening mammography for women 50 years and older since 2009.^9^ It should be noted that these same recommendations have also been adopted by the Federal Bureau of Prisons' clinical guidance on preventive healthcare screening.^10^ Unfortunately, overall, only 79% of women aged 50 years and older have received a screening mammogram within the past 2 years.^11^

Women in jail may also be underrepresented when it comes to breast cancer screening, and therefore they may be at increased risk for breast cancer disparities. A disproportionate number of incarcerated women are African American, thus potentially facing a similar disparity in mortality, although there is a paucity of research investigating incarcerated women's risk for breast cancer.^12^ Many of the disparities faced by American Americans are the same faced by incarcerated women, including low socioeconomic status, low health literacy, and poor access to continuous care. Incarcerated women also have other risk factors for developing breast cancer, including higher rates of poverty than the general population and behaviors associated with an increase in cancer breast risk, like alcohol and tobacco use.^13^ About half of our women report recent hazardous drinking, and nearly 80% of the jailed women in our sample smoke. Previous literature shows rates of up-to-date breast cancer screening for jailed women ranged from 41% to 58%.^12,14^ Both the previous studies were conducted with the breast cancer screening age as 40 years. No literature exists on rates of up-to-date screening mammography for U.S. women in jail since the updated USPSTF guidelines recommending the screening age to start at 50 years.

As the rate of women's incarceration grows faster than men, it is vital to address incarcerated women's health issues.^15^ To address this concern, our research team developed SHE, a Sexual Health Empowerment program, for women in jail. It is worth noting that jails, unlike prisons, house women with short lengths of incarceration—women awaiting sentencing, trial, or those with sentences for a year or less. Thus, this group of vulnerable women moves frequently between jails and communities. The original project focused on improving cervical health literacy and cervical cancer prevention given these realities. In SHE, we found that only 67% of these women were up-to-date on cervical cancer screening.^16^ With older literature showing low rates of up-to-date breast cancer screening in incarcerated women and our recent study finding less than ideal rates of up-to-date cervical cancer screening, it would not be unexpected that women in jail would continue to have low rates of up-to-date breast cancer screening. The objective of our study was to analyze data from the SHE project to determine what proportion of incarcerated women have up-to-date breast cancer mammography since the change in USPSTF recommendations for biennial screening mammograms starting at the age of 50 years. We also hope to address an important gap in the literature regarding breast cancer risk in a vulnerable population, thus leading to better understanding of this underrepresented group and the development of more targeted and relevant public health interventions.

## Materials and Methods

### Setting and intervention

The women were recruited from three jails in the Kansas City area that straddle both the Kansas and Missouri state line. Two of the jails were urban and have capacities for 300 and 800 people; the third jail was located within a suburb and has a capacity of 1000 people. About 15% of the total jail population was female. Two hundred sixty-one women who completed the baseline SHE survey from September 2014 to March 2016 were included in this secondary data analysis. SHE participants completed five 2-h long intervention sessions focused on increasing cervical health literacy and cancer screening. SHE was informed by social and feminist theory, designed to increase cervical health knowledge, reduce barriers encountered by personal beliefs of breast cancer, and improve self-efficacy and confidence navigating the healthcare system.^16,17^

### Data collection and analysis

The women completed a 158-question survey focused mainly on cervical cancer risks, prevention, and treatment.^16^ Two questions from the survey focused solely on breast health and were used for this analysis: “Have you ever had a mammogram” and “How long has it been since you had your last mammogram?” Women were considered “up-to-date” if they reported having had a mammogram within the past 2 years.^9^

Other demographic variables of interest were age, race, ethnicity, lifetime months incarcerated, jail location, education, employment status, intimate partner violence (IPV) within the past year, tobacco use within the previous 30 days, alcohol problems, and being up-to-date on cervical cancer screening (having had a Pap test in the past 3 years). Insurance status and having a personal doctor or medical home were included as these are variables that might affect the ease of obtaining a screening mammogram. Up-to-date cervical cancer tests were included because they are another screening test for women's health that may be correlated with being up-to-date on other types of women's health screenings. IPV was included as a variable because it is such a pervasive experience among this group of women and because of its association with other women's health variables in previous studies.^16,18^

Data analysis was performed using SAS software version 3.7. Univariate and tabulate procedures were used to obtain descriptive statistics on the overall sample and responses for main outcome variables. Bivariate chi-square procedures tested for associations between main outcome variables and independent variables. Odds ratios (ORs) were obtained from bivariate tests as well. If cell counts were <5 for chi-square tests, then the Fisher's Exact test was used to determine significant associations between independent variables and outcome variables. The independent variable, lifetime months incarcerated, was dichotomized based on the overall sample's median value of 7 months. For the outcome variable, up-to-date mammogram, only women 50 years old or older were included in analysis because of current USPSTF recommendations for mammogram screening. An alpha of <0.05 was considered significant for all calculations.

## Results

The secondary data analysis included 261 participants. Participant characteristics are shown in Table 1. About half of the participants were nonwhite (47.1%). Women reported being incarcerated for an average of 24.2 months over their lifetime (the median value of months incarcerated was 7.0). Most graduated from high school (62.4%), but were unemployed (59.7%) and lacked insurance before incarceration (59.4%). Over half of the women experienced IPV in the past year (61.3%).

**Table 1.** **Participant Characteristics, *N* = 261**

	*N* (%)
Age, mean (standard deviation)	33.7 (9.9)
Race	
White	128 (49.0)
Black	83 (31.8)
Other	40 (15.3)
Hispanic	21 (8.0)
Lifetime months incarcerated, median (IQR)	7.0 (21.0)
Jail	
Jackson County	112 (42.9)
Johnson County	50 (19.1)
Wyandotte County	99 (37.9)
Education	
Less than high school	88 (33.7)
High school or more	163 (62.4)
Employment	
Employed before incarceration	85 (32.5)
Unemployed before incarceration	156 (59.7)
Insured before incarceration	106 (40.6)
Has a personal doctor	103 (39.4)
Has a medical home	183 (70.1)
IPV in past year	160 (61.3)
Tobacco use in last 30 days	205 (78.5)
Alcohol problem^a^	132 (50.5)
Have ever had a clinical breast examination	199 (76.2)
Has ever had a mammogram	110 (42.1)
40–49 years old (*N* = 41)	28 (68.3)
50+ years old (*N* = 28)	21 (75.0)
Pap smear within past 3 years	174 (66.6)

^a^ Assessed using AUDIT-C, which is scored on a scale of 0–12 (scores of 0 reflect no alcohol use). In women, a score of 3 or more is considered positive for alcohol problems.^18^

AUDIT-C, Alcohol Use Disorders Identification Test-Concise; IPV, intimate partner violence.

### Ever had a mammogram

One hundred ten (42.1%) of all women had ever had a mammogram. Of the 28 women, 50 years old or older, 21 (75.0%) report ever having a mammogram. Overall, women in our cohort were significantly more likely to have ever had a mammogram if they were black compared with white or another race (55.4% vs. 33.6%, 43.5%, *p* < 0.01), had a personal doctor (OR = 1.8, 95% confidence interval [CI] 1.10–3.07), or medical home (OR = 1.8, 95% CI 1.00–3.25). Women ≥50 years old were significantly more likely to have ever had a mammogram if they were black compared with white or another race (100% vs. 53.8%, 50%, *p* = 0.01) or had a personal doctor (OR = 8.0, 95% CI 1.15–55.2) (Table 2).

**Table 2.** **Association Between Participant Characteristics and Ever Had a Mammogram**

	Ever had a mammogram, *N* = 261, *N* (%)			Ever had a mammogram, 50+ years old, *N* = 28, *N* (%)		
	Yes	No	*p*	Yes	No	*p*
Overall	110 (42.1)	145 (55.5)		21 (75.0)	7 (25.0)	
Race			**<0.01**			**0.01**
White	42 (33.6)	83 (66.4)		7 (53.8)	6 (46.1)	
Black	46 (55.4)	37 (44.5)		13 (100.0)	0 (0)	
Other	17 (43.5)	22 (56.4)		1 (50.0)	1 (50.0)	
Ethnicity			0.87			0.81
Hispanic	9 (45.0)	11 (55.0)		—	—	
Non-Hispanic	97 (43.1)	128 (56.8)		21 (75.0)	7 (25.0)	
Lifetime months incarcerated			0.48			0.82
≥7	61 (45.1)	74 (54.8)		13 (76.4)	4 (23.5)	
<7	49 (40.8)	71 (59.1)		8 (72.7)	3 (27.2)	
Education			0.68			0.58
Less than high school	35 (40.7)	51 (59.3)		3 (60.0)	2 (40.0)	
High school or more	69 (43.4)	35 (40.7)		16 (76.1)	5 (23.8)	
Employment			0.97			0.63
Yes	35 (41.6)	49 (58.3)		5 (71.4)	2 (28.5)	
No	63 (41.4)	89 (58.5)		16 (80.0)	4 (20.0)	
Insurance			0.80			0.66
Yes	44 (41.9)	61 (58.1)		8 (80.0)	2 (20.0)	
No	52 (40.3)	77 (59.6)		11 (68.7)	5 (31.2)	
Personal Doctor			**0.01**			0.07
Yes	53 (51.9)	49 (48.0)		13 (92.8)	1 (7.1)	
No	54 (36.9)	92 (63.0)		8 (57.1)	6 (42.8)	
Medical Home			**0.04**			**0.04**
Yes	85 (46.9)	96 (53.0)		18 (85.7)	3 (14.2)	
No	22 (32.8)	45 (67.1)		3 (42.8)	4 (57.1)	
IPV in past year			0.86			0.10
Yes	68 (43.3)	89 (56.6)		14 (87.5)	2 (12.5)	
No	40 (44.4)	50 (55.5)		7 (58.3)	5 (41.6)	
Tobacco use in the last 30 days			0.05			1.0
Yes	80 (40.0)	120 (60.0)		19 (73.0)	7 (26.9)	
No	27 (55.1)	22 (44.9)		2 (100.0)	0 (0)	
Alcohol problem^a^						
Yes	59 (45.3)	71 (54.6)	0.45	12 (80.0)	3 (20.0)	0.67
No	51 (40.8)	74 (59.2)		9 (69.2)	4 (30.7)	
Up-to-date Pap test (within past 3 years)			0.51			0.14
Yes	76 (44.1)	96 (55.8)		13 (92.8)	1 (7.1)	
No	28 (49.1)	29 (50.8)		8 (66.6)	4 (33.3)	

Bold indicates the significant *p*-values < 0.05.

^a^ Assessed using AUDIT-C, which is scored on a scale of 0–12 (scores of 0 reflect no alcohol use). In women, a score of 3 or more is considered positive for alcohol problems.^18^

### Up-to-date mammogram

Of the 28 women, 50 years or older, 11 (39.3%) have had a mammogram within the recommended past 2 years (Table 3). Women in this group were significantly more likely to have an up-to-date mammogram if they had experienced IPV within the past year (71.4% vs. 14.2%, *p* = 0.02) and if they had an up-to-date cervical cancer screening within the past 3 years (76.9% vs. 12.5%, *p* < 0.01). There was no significant association between up-to-date mammogram and race, ethnicity, lifetime months incarcerated, education, employment, insurance, personal doctor, or medical home.

**Table 3.** **Up-To-Date Mammogram (Within Past 2 Years) for Women At Least 50 Years Old, *N* = 28**

	*N* (%)
Ever had a mammogram	21 (75.0)
Last time you had a mammogram (*N* = 21)^a^	
Past year	5 (22.7)
Past 2 years	6 (27.3)
Past 3 years	3 (13.6)
Past 5 years	4 (18.2)
5 or more years	4 (18.2)

^a^ While 21 women indicated that they had ever received a mammogram, 22 women reported a timeframe, during which they received their last mammogram. This is attributed to self-report error.

## Discussion

Our secondary data analysis showed that while many (75.0%) women of 50 years old or older have received a mammogram, few (39.3%) were up-to-date. This is far less than the general population, in which 79% of women are up-to-date.^1^ We report up-to-date mammography rates in jailed women that are relatively unchanged from older literature published 8–13 years ago, in which 41–58% reported having up-to-date mammograms.^12,14^ Our study is possibly the first U.S. study to assess rates of up-to-date mammography in jailed women 50 years old and older since the USPSTF changed its guidelines to start screening from 40 to 50 years old. The continued low rates of up-to-date mammography in jailed women and stark contrast compared to the general population highlight the need for professional education regarding clinical and provider interventions focused on breast cancer prevention and access to care.

It is not unexpected that women with a personal doctor or medical home were more likely to have had a mammogram. Likewise, up-to-date cervical cancer screening was associated with an up-to-date mammography, which has also been shown by others.^19^ Interestingly, women who have experienced IPV in the past year were more likely to have an up-to-date mammogram. There is previous literature that shows that women with IPV have decreased odds of being up-to-date on mammography,^20^ however, other literature has shown that women who are abused are more likely than women who report no abuse to seek medical care for a myriad of concerns.^21–24^ It could be that because these women are frequenting their providers more, their providers have more opportunities to recommend screening mammography. This stresses the need for comprehensive care when this vulnerable group of women seeks medical care.

Our study also found a higher proportion of black women, compared with white women and women of other races, had ever had a mammogram. This is consistent with the Center for Disease Control and Prevention's Behavioral Risk Factor Surveillance System (BRFSS), which also reports higher rates of mammography in blacks (83.3%) compared with whites (77.6%) and other races (75.6%).^25^ Previous literature has shown lower rates of screening mammography among black women.^2,26^ Perhaps, the reason for this discrepancy is difference between screening and diagnostic mammograms. Our survey, along with the BRFSS, did not differentiate between screening and diagnostic mammograms. In the question to participants, both just asked “Have you ever had a mammogram?”^16,27^ Black women may get mammograms, but are often diagnosed at a later stage and with more severe disease and higher mortality because they do not get as many *screening* mammograms.^3–5^ Another possibility supported by our data is that black women get a mammogram once, but do not continue every 2 years, which is why race was not associated with up-to-date mammogram in our analysis.

Interestingly, many women <50 years old in our study reported having a mammogram. There is much controversy surrounding screening mammograms in this age group for average risk women. The USPSTF gives mammograms in women 40–49 years old a “Grade C,” which indicates that this is not an evidence-based best practice. While they do not recommend against mammography in this age group, they stress that there is no net benefit, and therefore must be based on the individual risk.^9^ Yet, the American Cancer Society has recommended yearly screening mammography for all women starting at age 45 since 2015, a change from the previous recommendation in 2003, which was yearly screening mammogram at age 40.^28^ These conflicting recommendations appear to cause confusion surrounding patients and physicians regarding screening guidelines in this age group, as screening mammography rates actually increased despite the 2009 USPSTF guideline change.^29^ A 2015 Veteran Affairs study showed that before a knowledge intervention, 82% of primary care providers advised all women 40–49 years old to undergo screening mammography, regardless of risk.^30^ Unfortunately, we do not know the reasons for mammography in this age group for our study. One potential explanation for higher rates of screening among younger women is that the Affordable Care Act includes a provision for annual screening mammography for women aged 40 and older, thus eliminating financial barriers for accessing care.^31^

Our study is not without limitations. Because SHE focused on cervical cancer literacy, few questions were specific to breast health/cancer, therefore, we do not have more detailed data focusing on breast cancer literacy and it is not known the reasons for mammography in this study population (screening vs. diagnostic). In addition, our data are based on patient report, not medical record review and may not be accurate. We know cervical health literacy is low in this group women, with women often misinterpreting Pap smear procedures.^32^ It would not therefore be unexpected that these women may not know what a mammogram is and if they truly have or have not had one. Although we did say, “A mammogram is an x-ray of each breast to look for breast cancer,” before asking the question in the survey. In addition, our sample size is ultimately too small to conclude that there are low rates of up-to-date mammography in all jailed women. However, our study joins a small but growing body of literature pointing to disparities in cancer screening, morbidity, and mortality for jailed women. Future research is needed in this area.

## Conclusion

Incarcerated women have increased risks for women's health problems compared with nonincarcerated women. Breast cancer risk among women with criminal justice histories may be another example of this. Based on our previous research showing low cervical health literacy and this secondary data analysis showing low rates of up-to-date screening mammograms, a jail-based breast cancer prevention intervention may be a next step to increase the uptake of screening mammography in this group.^17^ In addition, clinicians should continue to be aware of underrepresented groups at greatest risk for breast cancer morbidity and mortality, including the vulnerable population of incarcerated women.

## Abbreviations Used

BRFSS: Behavioral Risk Factor Surveillance System
CI: confidence interval
IPV: intimate partner violence
OR: odds ratio
SHE: Sexual Health Empowerment
USPSTF: United States Preventive Services Task Force
